# A National Study on the Comparative Burden of Pedestrian Injuries from Falls Relative to Pedestrian Injuries from Motor Vehicle Collisions

**DOI:** 10.21203/rs.3.rs-3218781/v1

**Published:** 2023-08-07

**Authors:** Andrew G. Rundle, Remle P. Crowe, Henry E. Wang, John R. Beard, Alexander X. Lo

**Affiliations:** Columbia University Mailman School of Public Health; ESO; The Ohio State University Wexner Medical Center; Columbia University Mailman School of Public Health; Northwestern University Feinberg School of Medicine

## Abstract

Pedestrian injuries from falls are an understudied cause of morbidity. Here we compare the burden of pedestrian injuries from falls occurring on streets and sidewalks with that from motor vehicle collisions.

Data on injurious falls on streets and sidewalks, and pedestrian-motor vehicle collisions, to which Emergency Medical Services responded, along with pedestrian and incident characteristics, were identified in the 2019 National Emergency Medical Services Information System database.

In total, 129,343 injurious falls and 33,910 pedestrians-motor vehicle collisions were identified, with 89% of the incidents occurring in urban areas. Thirty two percent of pedestrians struck by motor vehicles were coded as Emergent or Critical by Emergency Medical Services, while 20% of pedestrians injured by falls were similarly coded. However, the number of pedestrians whose acuity was coded as Emergent or Critical was 2.33 times as high for injurious falls as compared with pedestrians-motor vehicle collisions. This ratio was nearly double at 4.3 for individuals 50 years and older, and almost triple at 6.5 for those 65 years and older.

In conclusion, there has been substantial and appropriate policy attention given to preventing pedestrian injuries from motor vehicles, but disproportionately little to pedestrian falls. However, the population burden of injurious pedestrian falls is significantly greater and justifies an increased focus on outdoor falls prevention, in addition to urban design, policy and built environment interventions to reduce injurious falls on streets and sidewalks, than currently exists across the U.S.

## Introduction

The U.S. Department of Health and Human Services’ Healthy People 2030 Objectives include several goals to increase pedestrian activity and improve pedestrian safety.^1^ However, the vast majority of pedestrian safety research, pedestrian safety policy and built environment interventions have focused on pedestrian injuries from motor vehicles. There has been minimal focus on interventions to prevent injurious pedestrian falls that occur on streets and sidewalks, even though pedestrian injuries from falls and from motor vehicles occur in the same or adjacent environments. This balance of research and policy focus between these two types of pedestrian injury may be appropriate if it matches the respective distribution of mortality and morbidity in the population, yet evidence on the relative burden of injurious falls in these settings is lacking.

The negative impact of falls on the function and health of older persons, and the particular threat to healthy aging while living independently in their communities is well established.^2–6^ However, classical falls prevention doctrine, including fall prevention guidelines from the American Geriatric Society, British Geriatric Society and American Association of Orthopaedic Surgeons, has focused on person-level factors and omits the environment beyond the individual’s home, such as outdoor hazards.^2, 6–8^ Outdoor falls are often associated with health-promoting activities (e.g., walking, or running) and risk of falls is influenced by environmental factors and physical aspects of the built environment.^9, 10^

The National Highway Traffic Safety Administration (NHTSA) and the Centers for Disease Control and Prevention (CDC) have robust systems to track injuries and fatalities for pedestrians struck by motor vehicles. The NHTSA uses probability samples of police reports and in 2019 estimated that there were 76,000 pedestrians injured by motor vehicles and 6,205 deaths.^11^ The CDC uses data from a probability sample of hospitals and estimated that 136,314 visits to emergency departments (ED) for non-fatal injuries to pedestrians hit by motor vehicles and 6,681 for fatal injuries occurred in 2019.^12^ However, there is no equivalent surveillance system for tracking pedestrian falls by location.^12, 13^

Analyses of the 1997–2010 National Health Interview Survey (NHIS) data estimated that, among community dwelling adults, 518,000 fall injuries occurred annually outdoors on streets and sidewalks that required medical attention, though data on the severity of the injury or source of medical attention were lacking.^14^ Analysis of the 2019 National Emergency Medical Services Information System (NEMSIS) data identified 129,408 injurious falls that occurred on streets and sidewalks that required a response from Emergency Medical Services (EMS).^13^ The difference in burden of falls estimated from the NHIS and NEMSIS likely reflects the difference in the level of injury severity required for an incident to be included in the estimates: any medical attention for NHIS and an EMS activation for NEMSIS. While NHIS and NEMSIS data produce substantially different estimates of injurious falls on streets and sidewalks, both estimates are substantially larger than the estimates of pedestrian injuries from motor vehicles published by the NHTSA. The estimate from NEMSIS for falls on streets and sidewalks is on par with the CDC’s estimates of the number of pedestrians injured by motor vehicles and treated in hospitals. Thus, these disparate data suggest that the population burden of pedestrian falls on sidewalks and streets resulting in injury requiring emergency care is greater than the population burden of pedestrians injured by motor vehicles.

Our objective was to use a single data collection system, NEMSIS, to compare the burden of pedestrian injuries from motor vehicles to that of pedestrian falls occurring on streets and sidewalks that resulted in an EMS encounter. We also compared the severity of the injuries, the medical disposition of these patients and the descriptive epidemiology of these events.

## Methods

### Study Design and Data Source

We conducted a retrospective analysis using publicly available 2019 NEMSIS data (released 2021). NEMSIS is a program of the NHTSA Office of Emergency Medical Services and is the largest repository of EMS records in the U.S., with > 34 million events from over 10,000 EMS agencies.^15, 16^ NEMSIS data are released as a de-identified, Health Insurance Portability and Accountability Act exempt, publicly available dataset, hosted by the University of Utah with local Institutional Review Board (IRB) oversight (https://nemsis.org/using-ems-data/). Therefore, no further IRB review was sought for these analyses. We used the most recent NEMSIS dataset available prior to the COVID-19 pandemic, in order to avoid the well-documented impact of the COVID-19 pandemic and stay-at-home orders on walking behavior and the incidence of falls and traumatic injuries (https://www.cdc.gov/injury/wisqars/index.html)^17–19^ and on EMS response patterns, particularly with regards to traumatic falls and motor vehicle collisions.^20, 21^ We believe the 2019 NEMSIS data most accurately reflects the true patterns of pedestrian injuries exclusive of the influence of COVID-19.

### Inclusion of Records

Records were excluded if the Disposition (eDisposition.12) of the response was listed as Canceled (codes 4212007, 4212009, 4212011), Standby-No Services or Support Provided (code 4212039), or Transport Non-patient (code 4212043) or eResponse.05 was coded as Interfacility Transport (code 2205003) or Medical Transport (code 2205007).

### Measures

The identification of injurious falls and pedestrian injuries from motor vehicles builds upon our prior methods.^13^ Data from the NEMSIS eScene.09 variable was used to code the patient locations as “Outdoors – on a street or sidewalk”. The judgement of the EMS clinician on the cause of any injury suffered by the patient is documented via ICD10 codes in the NEMSIS eInjury.01 variable with multiple entries allowed per patient. The eInjury.01 data were used to identify injurious falls and pedestrians injured by motor vehicles.^13^ ICD10 codes in the V03 series were used to identify injuries to pedestrians caused by motor vehicles. As there can be multiple entries for eInjury.01 per patient there were some patients for whom the cause of the injuries was ambiguous because the eInjury.01 variable included both a code for an injury from a fall and a code for an injury from a motor vehicle collision. Fifty-nine patients whose injury location was coded as “Outdoors – on a street or sidewalk” had ambiguous eInjury.01 codes and were dropped from the analyses. The eSituation.13 variable reports the responding EMS clinicians’ rating of the patient’s condition on scene. EMS clinicians are trained to categorize patient acuity using nationally standardized definitions to categorize each patient as “Critical”, “Emergent” and “Lower Acuity”, based on the Patient Acuity Definitions defined by the NHTSA National EMS Core Content and which follows the Model of Clinical Practice of Emergency Medicine.^22^ The eSituation.13 variable adds a fourth category “Dead without Resuscitation Efforts” to these three acuity categories to rate the priorities for care in out-of-hospital settings. The eDisposition.12 variable indicated the patient’s final disposition, such as treated on scene, transported to hospital, or were dead on scene. As seizures can result in injurious falls, we excluded seizure events. If an ICD10 code for seizure was included in the eSituation.09 variable (patient’s primary symptom), or in the eSituation.11 or eSituation.12 variables which record the EMS clinician’s overall impression of the encounter, the patient was excluded.

### Sociodemographic Data

NEMSIS variables were used to categorize the age (ageyears), sex (ePatient.13), race and ethnicity (ePatient.14) of the patient. The CensusDivision variable was used to define the U.S. region where the incident occurred. The Urbanicity variables was used to define whether the incident occurred in Urban, Suburban, Rural or Wilderness environments.

### Statistical Analyses

Descriptive analyses were conducted of the EMS encounters for injurious falls and for pedestrians injured by motor vehicle collisions, restricted to events that occurred on streets and sidewalks. Graphical time series analyses were performed for injurious falls and pedestrian-motor vehicle collisions, and for the proportion of these two events that were pedestrian-motor vehicle collisions.

## Results

The 2019 NEMSIS dataset included data on 23,086,855 EMS responses where patient care was provided. Of these, for events occurring on streets or sidewalks, there were 129,343 injuries from falls and 33,910 injuries from motor vehicle collisions. Both injuries from falls (89%) and pedestrian injuries from motor vehicle collisions (91%) occurred predominantly in urban areas. Table 1 shows the sociodemographic characteristics of each injury type, the patient disposition and the patient acuity as rated by the EMS clinician. Notably, individuals age 50 years or greater comprised 62.4% of the patients treated for injurious falls, while they comprised only 33.1% of patients struck by motor vehicles and injured. Among patients struck by motor vehicles, 2.7% (n=925) died at the scene while among injurious fall patients the percentage was 0.1% (n=133). While the percentages of patients in the two injury groups who were transferred to another EMS professional or transported directly to a hospital were similar, the volume of patients was much higher for fall injuries: 108,378 patients injured by falls compared to 27,126 injured by motor vehicles. Similarly, while the percentage of pedestrians struck by motor vehicles whose acuity at scene was rated as Critical was higher than for patients who had experienced injurious falls, the actual numbers of patients were whose acuity was rated as Critical was similar between the two patient groups (Table 1). The number of fall patients whose acuity was classified as Emergent was substantially higher compared to patients struck by motor vehicles. Table 2 provides data on patient acuity by age group for pedestrians who experienced injurious falls and injuries from motor vehicles. Individuals age 50 years or older comprise the majority of pedestrians treated for falls occurring on streets and sidewalks. Overall across all age groups, the number of pedestrians whose acuity level was coded as either Emergent or Critical was 2.33 times as high for injurious falls as compared with the number of pedestrians with injuries from being struck by motor vehicles. This ratio was nearly double at 4.3 for pedestrians 50 years and older, and almost triple at 6.5 for those 65 years and older.

Figure 1 presents the time course of pedestrian falls and pedestrians injured by motor vehicles by hour of the day for weekend and weekdays and the percentage of these two injuries contributed by injuries from motor vehicles. The graphs depict the higher burden of injurious pedestrian falls overall for both weekdays and weekend days. Figure 2 shows a spike, as a percentage of injuries, for pedestrian injuries caused by motor vehicles during the weekday morning rush hour and then a rise from mid-afternoon into the evening. However, the maximum for the percentage of these two injuries caused by motor vehicles peaks at 35.8%.

## Discussion

The volume of EMS responses for injurious falls on streets and sidewalks was substantially greater than for pedestrians injured by motor vehicles. Similarly, the overall number of injured pedestrians with Emergent or Critical acuity rated by EMS was much higher for pedestrians with injurious fall than for pedestrians struck by motor vehicles. This is particularly true for individuals ≥ 50 years old, where the number of pedestrian injuries coded as Emergent or Critical acuity was 4.3-fold for falls than for pedestrians struck by motor vehicles. Although fewer in numbers, a larger proportion of pedestrians struck by motor vehicles were rated as Emergent or Critical acuity and had a higher probability of being dead on scene compared to pedestrians with an injurious fall on streets or sidewalks. The vast majority of both types of injury occur in urban spaces, suggesting that urban design, policy and built environment interventions are important tools for reducing morbidity and mortality.

The overall number of older pedestrians who fell and required EMS responses is alarming, especially the number coded as Emergent or Critical on scene by EMS.^23^ Unintentional injuries rank as the 8th cause of death, and fall-related injuries account for 80% of all trauma admissions among older persons in the U.S.^24^ Even falls without significant injuries increase the risk of declines in mobility and social participation.^25^ Falls without injuries are also associated with the fear of falling, a well-described phenomenon among older persons with no clear modifiable risk factor,^26^ but compounds the detrimental effects of falls by restricting healthy activities^27^ and increasing the incidence of disability.^28^

Despite the high incidence of pedestrians experiencing injurious falls, there has been much less policy attention given to this public health issue than to preventing pedestrian injuries from motor vehicles. We argue that this likely arises from differences in who is responsible for, and who pays for, sidewalk and road maintenance.^29^ In many cities landowners are responsible for the maintenance of sidewalks that are along the perimeter of the land parcel, both for snow and ice removal and for repairing damage to the sidewalk surface.^29^ Thus, a single city block can vary tremendously in terms of the cleanliness and maintenance of the physical surface of the sidewalk. Roadways, however, are maintained by city, county and state agencies, organizations likely to have better access to human and material resources to maintain infrastructure than individual property owners. Moreover, maintenance of roadbed surfaces tends to focus on larger scale damage (e.g. potholes) that might interfere with driving, rather than smaller hazards that might pose a risk to pedestrians crossing the street. Some cities provide financial incentives to homeowners to fix physical damage to sidewalks and/or provide hotlines to report damaged sidewalks so that cities can serve notice or fines to landowners.^13^ It seems possible that when municipal work crews are dispatched to repair roadways, install curb extensions, plant street trees, or maintain medians, these same crews can also repair sidewalks along that roadway.

Many cities have robust surveillance programs for motor vehicle crashes and injuries to pedestrians and cyclists from motor vehicles. They also have an arsenal of policy, design, and built environment interventions to increase motor vehicle related road safety, with much of the work to develop these interventions having been done by the NHTSA, the Vision Zero program and the Safe Routes to School program.^30, 31^ However, there is a lack of robust surveillance systems for monitoring pedestrian falls occurring on sidewalks and roadbeds.^32^ Without such systems it is difficult to understand the burden of falls and motivate the development of prevention programs or prioritize interventions programs to high-risk areas. We have argued that the NEMSIS system of reporting EMS activations presents an opportunity for states or cities to develop such a surveillance system based on standardized EMS data.^13^

Creating urban environments that support the health and engagement of older persons is becoming increasingly important as populations age. Multiple characteristics can contribute to making a pedestrian environment “age-friendly” including walkable design, ambient temperature, lighting, signage, appropriate street crossing design or crossing speeds and provision of seating.^33^ But pedestrian safety is also critical and requires proper maintenance of sidewalks.^33^ Designing an age-friendly street environment is therefore not a straightforward task and involves many trade-offs. For example, street trees create welcoming and shadier environments that encourage people to leave their homes and be physically active. The shade they provide reduces ambient temperatures on streets and two recent studies suggest that lower outdoor temperatures are associated with lower risk for pedestrian falls among older adults.^34, 35^ However, the roots of poorly chosen, inappropriately placed or poorly maintained trees can disrupt pavements, and fallen leaves or branches can create trip hazards that increase the risk of falls.^36^ An appropriate policy response is further complicated by the disjointed responsibility for road and sidewalk maintenance. The burden of injurious falls among older pedestrians highlighted by this paper suggests new approaches are required that span all aspects of age-friendly design. It is likely that, rather than relying on individual property owners, more centralized mechanisms for sidewalk maintenance are required.^29^

The NEMSIS data recorded fewer pedestrian injuries and fatalities from motor vehicle collisions than reported by the NHTSA or the CDC WISQARS. The differences in the totals across the three systems may reflect the differences in data gathering: Administrative reports of EMS activations with fatalities counted only for those dead at the scene (NEMSIS); a sample of police reports for injuries and all fatalities within 30 days of a collision (NHTSA); and a probability sample of hospitals (CDC). It is also possible that not all police-reported collisions or ED visits involve an EMS response. For instance, among the 15,221 pedestrians injured by motor vehicles recorded in NY State Department of Motor Vehicles data for 2019, 60% of the pedestrians were described as having minor injuries and thus perhaps EMS activations for these collisions did not involve treatment or transport.^37^ Another weakness of the NEMSIS data is that injury acuity data were missing for 27% of the fall injuries and 31% of the motor vehicle injuries. However, even if all of the pedestrians injured by motor vehicles with missing acuity ratings had the three worse acuity ratings (Emergent, Critical or Dead without Resuscitation Efforts), there would still be fewer pedestrians injured by motor vehicles than pedestrians injured by falls whose acuity was classified into these categories. Lastly, the NEMSIS data does not differentiate between events occurring on streets versus sidewalks, but we can logically assume, for events coded as occurring on streets or sidewalks, that the pedestrians struck by motor vehicles were more likely to be struck in the street than on the sidewalk. Analyses of 1997–2010 NHIS data showed that 38.4% of pedestrian falls occurred on sidewalks, 21.4% at the curb and 40.2% on streets. However, it is unclear whether these percentages for falls requiring any medical attention can be applied to falls that EMS responded to. The strengths of NEMSIS are that it covers both pedestrian falls and injuries from motor vehicles in a single data set with a single system for gathering data and provides consistent coding for the location of the event, disposition of the patient and patient acuity allowing for head-to-head comparisons.

## Conclusion

These findings suggest that while the probability of a pedestrian suffering a severe injury is higher for motor vehicle collisions than for falls, the population burden of the total number of injuries, and of severe injuries, from falls is significantly higher. This is particularly true for individuals 50 years or older. From a public health perspective, the relatively stronger policy focus on preventing pedestrian injuries from motor vehicles compared to preventing injurious falls on streets and sidewalks is disproportionate with the population morbidity burden. While maintaining our focus on preventing pedestrian injuries from motor vehicles, policy makers and public health practitioners should increase their focus on pedestrian safety from falls. This will involve efforts to develop surveillance systems, to reconsider the responsibility for sidewalk maintenance, to improve the implementation of age friendly cities, and to develop creative programs to combine roadbed and sidewalk maintenance.

## Figures and Tables

**Table 1.** Descriptive Statistics for Injured Pedestrians

	Pedestrian Injured by fall or motor vehicle	
	Fall Injury N (Column %)	Injury from motor vehicle N (Column %)
Total	129,343	33,910
Patient Sex		
Male	74,667 (58)	20,484 (60)
Female	54,052 (42)	13,214 (39)
Not Recorded	624 (0)	212 (1)
Patient Age		
<21	8,984 (7)	6,765 (20)
21 – 29	11,179 (9)	5,586 (16)
30-39	13,539 (10)	5,473 (16)
40-49	14,177 (11)	4,415 (13)
50-64	36,417 (28)	7,181 (21)
65+	44,270 (34)	4,047 (12)
Not Recorded	777 (1)	443 (1)
Patient Race and Ethnicity		
American Indian or Alaska Native	601 (0)	156 (0)
Asian	898 (1)	232 (1)
Black or African American	14,183 (11)	3,668 (11)
Hispanic or Latin	5,024 (4)	1,278 (4)
Native Hawaiian or Other Pacific Islander	169 (0)	53 (0)
White	40,248 (31)	10,757 (32)
Mixed Race	73 (0)	30 (0)
Not Recorded	68,147 (53)	17,736 (52)
Urbanicity		
Urban	114,480 (89)	30,843 (91)
Suburban	4,718 (4)	941 (3)
Rural	5,340 (4)	955 (3)
Wilderness	1,600 (1)	213 (1)
Not Recorded	3,305 (3)	958 (3)
Census Division		
East North Central	25 (0)	13 (0)
East South Central	12,422 (10)	2,314 (7)
Middle Atlantic	3,664 (3)	1,109 (3)
Mountain	9,508 (7)	3,003 (9)
New England	16,583 (13)	3,794 (11)
Pacific	5,645 (4)	1,205 (4)
South Atlantic	25,823 (20)	8,181 (24)
West North Central	31,242 (24)	8,689 (26)
West South Central	8,280 (6)	1,385 (4)
Not Reported	16,151 (12)	4,217 (12)
Disposition of the EMS Activation		
Patient dead at scene	133 (0)	925 (3)
Patient evaluated, no treatment or transport required	2,929 (2)	1,079 (3)
Patient refused evaluation	5,766 (4)	1,368 (4)
Patient treated and released AMA	7,708 (6)	2,058 (6)
Patient treated and released per protocol	2,395 (2)	754 (2)
Patient treated and transferred or transported	108,378 (84)	27,126 (80)
Other	2,034 (2)	600 (2)
EMS Clinician Rating of Patient Acuity		
Lower Acuity (Green)	68,811 (53)	11,958 (35)
Emergent (Yellow)	22,512 (17)	7,234 (21)
Critical (Red)	3,029 (2)	3,725 (11)
Dead without Resuscitation Efforts (Black)	91 (0)	468 (1)
Not Reported	34,900 (27)	10,525 (31)

Abbreviations: AMA, Against Medical Advice; EMS, Emergency Medical Services.

**Table 2.** Emergency Medical Services Clinician Rating of Patient Acuity by Patient Age

		Pedestrian Injured by fall or motor vehicle	
Patient Age	Patient Acuity	Fall Injury N (Column %)	Injury from motor vehicle N (Column %)
<21	Lower Acuity (Green)	5,018 (56)	2,730 (40)
	Emergent (Yellow)	1,367 (15)	1,445 (21)
	Critical (Red)	216 (2)	568 (8)
	Dead without Resuscitation Efforts (Black)	4 (0)	24 (0)
	Not Reported	2,379 (26)	1,998 (30)
21 - 29	Lower Acuity ( Green)	6,161 (55)	2,087 (37)
	Emergent (Yellow)	1,771 (16)	1,172 (21)
	Critical (Red)	282 (3)	548 (10)
	Dead without Resuscitation Efforts (Black)	11 (0)	55 (1)
	Not Reported	2,954 (26)	1,724 (31)
30-39	Lower Acuity (Green)	7,298 (54)	1,861 (34)
	Emergent (Yellow)	2,254 (17)	1,210 (22)
	Critical (Red)	350 (3)	651 (12)
	Dead without Resuscitation Efforts (Black)	16 (0)	97 (2)
	Not Reported	3,621 (27)	1,654 (30)
40-49	Lower Acuity (Green)	7,480 (53)	1,548 (35)
	Emergent (Yellow)	2,418 (17)	907 (21)
	Critical (Red)	367 (3)	509 (12)
	Dead without Resuscitation Efforts (Black)	15 (0)	94 (2)
	Not Reported	3,897 (27)	1,357 (31)
50-64	Lower Acuity (Green)	19,731 (54)	2,407 (34)
	Emergent (Yellow)	6,196 (17)	1,529 (21)
	Critical (Red)	808 (2)	856 (12)
	Dead without Resuscitation Efforts (Black)	17 (0)	111 (2)
	Not Reported	9,665 (27)	2,278 (32)
65+	Lower Acuity (Green)	22,845 (52)	1,286 (32)
	Emergent (Yellow)	8,373 (19)	932 (23)
	Critical (Red)	972 (2)	495 (12)
	Dead without Resuscitation Efforts (Black)	26 (0)	50 (1)
	Not Reported	12,054 (27)	1,284 (32)
Not Recorded	Lower Acuity (Green)	278 (36)	39 (9)
	Emergent (Yellow)	133 (17)	39 (9)
	Critical (Red)	34 (4)	98 (22)
	Dead without Resuscitation Efforts (Black)	2 (0)	37 (8)
	Not Reported	330 (42)	230 (52)
